# Morphology and genetic variation in the endangered tortoise *Manouria emys*: distinct lineages or plastron anomalies?

**DOI:** 10.1080/23802359.2018.1437795

**Published:** 2018-02-09

**Authors:** Shantanu Kundu, Vikas Kumar, Boni Amin Laskar, Kaomud Tyagi, Kailash Chandra

**Affiliations:** a Molecular Systematics Division, Centre for DNA Taxonomy, Zoological Survey of India, Kolkata, India;; b Freshwater Biology Regional Centre, Zoological Survey of India, Hyderabad, India

**Keywords:** Land tortoise, Pectoral scutes, mitochondrial DNA, Northeast India

## Abstract

Anecdotal reports indicate the allopatric populations of two *Manouria emys* subspecies differ in colour variation and plastron pattern, intimating that these may constitute separate evolutionary lineages. We examined the studied taxa both morphologically and genetically to determine the described morphological differences correlated with genetic divergence. Based on the plastron pattern, the study detected three morphologically different *M. emys* having their footmarks in northeast India, bordering international boundaries of Bangladesh and Myanmar. Nevertheless, we found shallow genetic divergence in both mtCOI and mtCytb gene segment among the different *M. emys* morphospecies. This study concludes that the detected plastron anomalies in *M. emys* do not suggest their distinct genetic lineages.

## Introduction

The biogeographic conditions in northeast India mostly correspond with that in Southeast Asia, and belong to the broad Indo-Malayan realms. The region is important with a remarkably higher concentration of land vertebrates offering scope for investigating species diversity (Datta-Roy et al. 2014). Among the 16 genera of land tortoise comprising 49 extant species, three genera, *Manouria*, *Geochelone* and *Indotestudo*, are distributed from northeast India to mainland Asia. The genus *Manouria* is composed of two species, *M. emys* and *M. impressa*; of which, *M. emys* is considered as the fourth largest land tortoise in mainland Asia (Schaffer and Morgan 2002; Stanford et al. 2015). Following its original description, taxonomic studies on this species have recognized the presence of two subspecies in it, *M. emys phayrei* and *M. emys emys*. *Manouria emys phayrei* is generally darker in colour with a dark brown, olive or black domed carapace; while, *M. emys emys* has a lighter yellowish brown, and flattened carapace (Moll 1989). A key difference between the two subspecies is that pectoral scutes meet with the plastral midline in *M. emys phayrei*, but widely separated in *M. emys emys* (Ernst and Barbour 1989; Stanford et al. 2015), which is however debatable.

The distribution pattern of these two subspecies is fascinating. *Manouria emys phayrei* is distributed from central and northern Thailand, Myanmar, and Bangladesh to India; while, *M. emys emys* is distributed from southern Thailand, Malaysia, Sumatra, Borneo, to some parts of the Indonesian Islands. *M. emys emys* is considered as the ‘southern subspecies’ and *M. emys phayrei* as the ‘northern subspecies’. The distribution of these two subspecies is separated at the tectonic side fault area, the Surat Thani gap, but exhibiting an intergradation in southern Thailand (Schaffer and Morgan 2002). Limited field survey and anecdotal evidence indicate that allopatric population of the endangered *M. emys* differ in size and their pectoral scutes, suggesting that these may constitute recently diverged distinct lineages. Earlier studies have discussed their genetic relatedness based on mitochondrial cytochrome b (mtCytb) region (Le et al. 2006); however, the genetic diversity at the inter-subspecies level on mitochondrial cytochrome oxidase I (mtCOI) gene, the agreed upon DNA barcode segment, was not characterized. Here, we investigated the genetic divergence based on two mitochondrial markers (mtCOI and mtCytb) in three morphological forms of *M. emys* across its international boundaries in northeast India.

## Materials and methods

### Taxon sampling and laboratory analysis

The specimens of *M. emys* subspecies were collected from northeast India and the bordering land of neighbouring countries, Myanmar and Bangladesh. A few individuals were also collected from pet keepers and their collection localities are unknown. Morphology was recorded following the original description as well as subsequent re-descriptions and taxonomic reviews (Ernst and Barbour 1989; Schaffer and Morgan 2002; Stanford et al. 2015). To avoid the risk of handling live animals for genetic investigation, we used cloacal swabs as a source of DNA. The methodological approach was approved by the Zoological Survey of India (ZSI), Ministry of Environment Forest and Climate Change (MoEF&CC) referring to the office memorandum No. F.223-81/2016/Tech./12769 and Science & Engineering Research Board (SERB), Department of Science and Technology (DST), Govt. of India referring to the letter no. F. No. PDF/2015/000302.

Genomic DNA was extracted following the QIAamp DNA Mini Kit standard protocol (Kundu et al. 2016) and stored at Centre for DNA Taxonomy, ZSI, Kolkata. We amplified partial mtCOI gene segment using the primer pair mentioned in Ward et al. (2005), and partial mtCytb using the primer pair mentioned in Verma and Singh (2003). The 25μl PCR mixture contains 10 pmol of each primer, 100 ng of DNA template, 1× PCR buffer, 1.0–1.5 mM of MgCl_2_, 0.25 mM of each dNTPs, and 0.25 U of Platinum Taq DNA Polymerase High fidelity (Invitrogen, Life Science Technologies, Carlsbad, CA) in a Veriti® Thermal Cycler (Applied Biosystems Inc., Foster City, CA). The PCR products were purified using QIAquickR Gel extraction kit (QIAGEN Inc., Germantown, MD), and cycle sequencing products were cleaned by using standard BigDye X-Terminator Purification Kit (Applied Biosystems Inc., Foster City, CA). A 48 capillary array 3730 DNA Analyzer (Applied Biosystems Inc., Foster City, CA) was used for bidirectional sequencing at the ZSI in-house sequencing facility.

### ESUs estimation and phylogenetic analyses

The chromatograms of both forward and reverse strands were checked and the noisy parts were trimmed at both the ends. The nucleotide BLAST (BLASTn) program was used to evaluate the sequences. The screened fragments were aligned using ClustalX software (Thompson et al. 2002) and finally, the sequences were compared in NCBI through BLASTn and ORF finder to examine the complete alignment and stop codons (http://www.ncbi.nlm.nih.gov/gorf/gorf.html). Primarily, the developed sequences were identified at the online identification web interfaces, BLASTn and BOLD Identification System (BOLD-IDs). The generated mtCOI sequences were analyzed through phylogeny and Kimura 2 parameter (K2P) genetic distance, while the generated mtCytb sequences were analyzed for K2P genetic distance and TCS networking of haplotypes with publically available database sequences. The Automatic Barcode Gap Discovery (ABGD) was performed for the studied dataset at the web interface (http://www.abi.snv.jussieu.fr/public/abgd/, web version March 2017); using a default value of relative gap width (*X* = 1.5) and K2P substitution model with other defaults parameters (Puillandre et al. 2012). Further, we applied the Bayesian implementation of Poisson tree processes model (bPTP) analysis to infer the Evolutionary Significant Units (ESUs) on a given phylogeny. The bifurcated phylogeny was inputted on the bPTP online server (http://species.h-its.org/ptp/, web version November 2016) using parameters: MCMC, 100000 generations; Thinning, 100; Burn-in, 0.1; Seed, 123 (Tang et al. 2014). The mean genetic divergences were calculated using K2P in MEGA6.0 (Tamura et al. 2013). Two phylogeny were constructed under the optimality criteria of neighbour-joining (NJ) in PAUP* 4.0b10 (Swofford 2002) with 1000 bootstrap support and Bayesian analysis (BA) using MrBayes 3.2 (Ronquist et al. 2012). For BA, Markov Chain Monte Carlo (MCMC) was performed with four chains for 1,000,000 generations, with trees sampled every 100 generations (the first 1000 trees were discarded as ‘burn in’). MCMC analysis was stationary when the maximum standard deviation of split frequencies reached below 0.01 and potential scale reduction factor (PSRF) approached 1.0. Further, to investigate the flow of genes, we used the haplotype sharing and TCS network as implemented in POPART (Leigh and Bryant 2015) with the mtCytb dataset.

## Results

**Figure 1.**
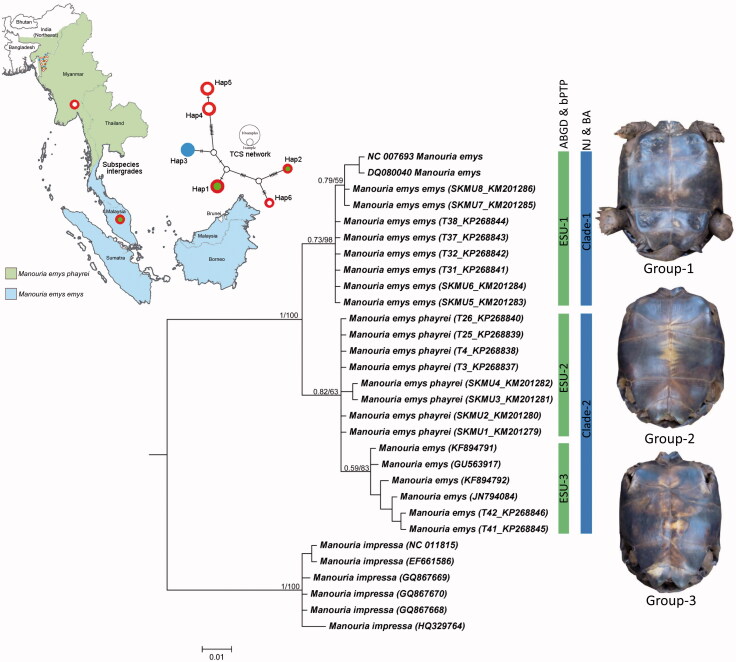
Map showing the distribution pattern of two subspecies and their intergradation zone in southern Thailand. Sampling sites indicated by dots. POPART generated TCS network for mtCytb haplotypes. Vertex with red-green shows Group 1, *M. emys emys* haplotypes, vertex with red-white shows Group 2 *M. emys phayrei* haplotypes and vertex with blue colour shows Group 3 haplotype. Bayesian phylogeny on mtCOI with posterior probability and bootstrap value by NJ analysis are superimposed with each node. The green bar represents the ESUs generated by ABGD and bPTP analysis while blue bar represents the clades generated by NJ and BA phylogenetic analysis. Plastron pattern of three morphological forms of *M. emys* shows with respective clades.

The study is based on the data from 18 specimens identified as *M. emys* in the size group of >47 cm in male and >45cm in female. The plastrons in the specimens were detected with three patterns of pectoral scutes. The pectoral scutes in eight specimens do not meet with the plastral midline which is apparent in the southern subspecies, *M. emys emys*, which we considered as Group 1. In other eight specimens, the inner edges of the anterior and the posterior margins of the pectoral scutes independently meet with the plastral midline which is apparent in the northern subspecies, *M. emys phayrei*, which we considered as Group 2. In rest two specimens, the inner edges of the anterior and the posterior margins of the pectoral scutes form a cone shape that meets with the plastral midline apparently presenting a complex form, which we considered as Group 3 (Figure 1). The morphology was compared with the type specimens of *M*. *emys* in the National Zoological Collection's (NZC), Kolkata, India. The specimens ZSI-813 and ZSI-814a from NZC possessed a pattern of pectoral scutes that is apparent in *M. emys phayrei*. The specimen ZSI-15492 was not in good condition for morphological study.

The BLASTn and BOLD-IDs revealed definitive identity matches (99–100%) for all the generated sequences. A total of 18 mtCOI and eight mtCytb sequences were generated in this study. Further, to obtain the cladding as robust as possible, six mtCOI sequences of *M. emys* were accessed from GenBank database (Table 1). A workout with mtCOI dataset on ABGD web interface showed a maximum of three initial as well as recursive partition at 0.0046 prior intraspecific divergences (*P*) and K2P substitution model. Further, the bPTP analysis with the same dataset also depicted three evolutionary significant units (ESUs). The specimens in ESU-1, ESU-2, and ESU-3 of ABGD and bPTP correspond with the specimens that were morphologically categorized into Group 1, Group 2, and Group 3, respectively. The highest K2P divergence using mtCOI dataset was 0.5% in ESU-1, 0.3% in ESU-2, and 0.7% in ESU-3. The divergence between ESU-1 and ESU-2 was 1.7%, ESU-1 and ESU-3 was 2%, and ESU-2 and ESU-3 was 0.8%. The mtCytb dataset was developed using eight generated sequences and two sequences from the database (DQ497315 for *M. emys emys* and DQ497316 for *M. emys phayrei*). The highest K2P divergence using mtCytb dataset was 0.9% in ESU-1, 0.02% in ESU-2, and 0% in ESU-3. The divergence between ESU-1 and ESU-2 was 2.5%, ESU-1 and ESU-3 was 2%, and ESU-2 and ESU-3 was 0.07%. The inter-species genetic divergence between *M. emys* and *M. impressa* was 9.4–10.6% in mtCOI and 7.8–8.9% in mtCytb gene.

**Table 1.** Information details of the studied *M*. *emys* collected from wild and pet trade in northeast India.

Species/subspecies	Voucher repository	GenBank accession mtCOI	GenBank accession mtCytb	Specimens locality	Haplotype of mtCytb
*M. e. emys*	SKMU5	KM201283	–	Pet kept	–
*M. e. emys*	SKMU6	KM201284	–	Pet kept	–
*M. e. emys*	SKMU7	KM201285	–	Pet kept	–
*M. e. emys*	SKMU8	KM201286	–	Pet kept	–
*M. e. emys*	T31	KP268841	–	23.75 N 92.85 E	–
*M. e. emys*	T32	KP268842	–	23.91 N 92.48 E	–
*M. e. emys*	T37	KP268843	KR014225	23.74 N 92.72 E	Hap1
*M. e. emys*	T38	KP268844	KR014224	23.75 N 92.85 E	Hap1
*M. e. emys*	AMCC157827	–	DQ497315	Le et al. (2006)	Hap2
*M. e. phayrei*	SKMU1	KM201279	–	Pet kept	–
*M. e. phayrei*	SKMU2	KM201280	–	Pet kept	–
*M. e. phayrei*	SKMU3	KM201281	–	Pet kept	–
*M. e. phayrei*	SKMU4	KM201282	–	Pet kept	–
*M. e. phayrei*	T3	KP268837	KR014231	23.75 N 92.85 E	Hap4
*M. e. phayrei*	T4	KP268838	KR014230	23.71 N 92.85 E	Hap4
*M. e. phayrei*	T25	KP268839	KR014229	23.73 N 92.71 E	Hap5
*M. e. phayrei*	T26	KP268840	KR014228	23.73 N 92.73 E	Hap5
*M. e. phayrei*	AMCC157828	–	DQ497316	Le et al. (2006)	Hap6
*M. emys*	T41	KP268845	KR014223	23.72 N 92.71 E	Hap3
*M. emys*	T42	KP268846	KR014222	23.71 N 92.72 E	Hap3
*M. emys*	AUTK73	KF894792	–	Database	–
*M. emys*	AUTK63	KF894791	–	Database	–
*M. emys*	AUTK8	JN794084	–	Database	–
*M. emys*	–	GU563917	–	Database	–
*M. emys*	–	NC_007693	–	Database	–
*M. emys*	–	DQ080040	–	Database	–

Occurrence and frequency of haplotypes in samples of *Manouria*. Voucher code with ‘SKMU’ and ‘T’ represents the samples collected from wild as well as kept as pet in northeast India and voucher code with ‘AMCC’ represents the samples from southeast Asia (Le et al. 2006).

The NJ and BA phylogeny revealed two clades: Clade 1 with two database sequences (NC_007693 and DQ080040) along with the generated sequences that correspond to specimens in ESU-1 and Group 1 as above, Clade-2 with four database sequences (KF894791, KF894792, GU563917, and JN794084) along with the generated sequences that correspond to specimens in ESU-2, ESU-3, Group 2 and Group 3. However, Clade 2 showed very shallow genetic divergence as above. Hence, the inspection in Group 2 and Group 3 becomes questionable and thought to be same taxa. The TCS network depicted a total of six haplotypes in the mtCytb dataset. The generated sequences of Group 1 specimens were contained in Hap1, Group 2 specimens were contained in Hap4 and Hap5, and Group 3 specimens were contained in Hap3. Hap2 and Hap6 were represented in each by a single database sequence from the native locality in Southeast Asia. Based on the haplotype grouping, it is evident that, *M. emys emys* and *M. emys phayrei* specimens studied from northeast India are distinct from Southeast Asian specimens with no sharing of haplotypes (Figure 1).

## Discussion

The phenotypic anomalies with regard to shell structure and appearance often create taxonomic confusion to identify Testudines species (Velo-Antón et al. 2011; Kundu et al. 2013). A previous study (Ernst and Barbour 1989) stated that hatchlings of both subspecies of *M*. *emys* appear similar, differing primarily in colour and pectoral scute arrangement. Differences become more pronounced during subsequent years when the carapace of *M*. *e*. *phayrei* develops a pronounced scute dimpling, while those of *M*. *e*. *emys* remain unchanged (Schaffer and Morgan 2002; Stanford et al. 2015). We observed three patterns of pectoral scute arrangement in adult specimens. In contrary to the previous study, we further observed a variation in pectoral scute arrangement in neonatal specimens of *M*. *e*. *phayrei*. The pectoral scute arrangement remains persistent throughout from hatchling to adult while the previous study documented progressive dimpling of scute in an individual from its hatchling to its subsequent life.

The analyses of mtCOI sequence data in NJ and BA support only two distinct clades in the dataset. The Clade 1 is presumably identified based on distribution information to be *M*. *e*. *emys* and the Clade 2 to be *M*. *e*. *phayrei*. Like the previous study, the Clade 1 and Clade 2 specimens possessed low genetic divergence within the clade suggesting the adequate flow of gene ignoring the phenotypic variation in terms of pectoral scutes arrangement. The previous study showed 1.3% genetic divergences in mtCytb gene between *M. emys emys* and *M. emys phayrei* (Le et al. 2006) while our study depicted genetic divergence of 1.7% in mtCOI and 2.5% in mtCytb which are moderately low than the congeneric genetic distance between *M. emys* and *M. impressa*. It has been shown that the distinct taxa are capable of maintaining largely discrete gene pools, allowing them to occur together in a widely overlapping distribution ranges as described on Box turtle (Fritz and Havaš 2014). Although, the other species delimitation methods (ABGD and bPTP) showed three different groups within the studied *M. emys* specimens, the study relies on the previous taxonomic description and accordance with present phylogenetic analysis (NJ and BA) to interpret their taxonomic question. The observed fact is that both the subspecies occurring in contiguous localities; far away from their intergradation zone, maintain shallow genetic divergence across inter-subspecies level and no sharing of haplotypes with the Southeast Asian population. This suggests that a natural migration or human-mediated transportation of southern subspecies, *M. emys emys* through trading. Notably, a recent study detected the existence of three non-native turtle species in the north-eastern region of India (Kundu et al. 2016). However, the extensive survey from wide geographical locations, reviewing their reproductive biology, effects of environmental factors in natural populations, and investigation through additional nuclear markers would be useful to understand the complex origin of phenotypic anomalies in natural populations (Velo-Antón et al. 2011). In summary, the study does not find any correlation between the different pattern of pectoral scutes and significant genetic divergence, and thus no evidence of genetically distinct lineages.
